# Hollow Silica Microparticles Based on Amphiphilic Polyphosphazenes

**DOI:** 10.3390/ma15144763

**Published:** 2022-07-07

**Authors:** Yolanda Salinas, Vanessa Poscher, Oliver Brüggemann, Ian Teasdale

**Affiliations:** 1 Institute of Polymer Chemistry, Johannes Kepler University Linz, Altenberger Straße 69, 4040 Linz, Austria; v.poscher@gmail.com (V.P.); oliver.brueggemann@jku.at (O.B.); ian.teasdale@jku.at (I.T.); 2 Linz Institute of Technology, Johannes Kepler University Linz, Altenberger Straße 69, 4040 Linz, Austria

**Keywords:** silica-based hollow microparticles, polyphosphazenes, thiol-ene photoreaction, hybrid materials, surfactant-free

## Abstract

Hollow microparticles are important materials, offering a larger surface area and lower density than their solid counterparts. Furthermore, their inner void space can be exploited for the encapsulation and release of guest species in a variety of applications. Herein, we present phosphazene-based silica hollow microparticles prepared via a surfactant-free sol-gel process through self-assembly of the alkoxysilyl-containing polymer in water–ethanol solution. Solely, a silane-derived polyphosphazene was used as the precursor for the microparticle formation, without additional classical silica sources. These novel hollow silica-based microparticles were prepared without surfactant, using a designed amphiphilic polyphosphazene for the particle formation made by two components, a hydrophilic unit consisting of 3-mercaptopropyl(trimethoxysilane), and a hydrophobic unit (dodecanethiol) attached to the double bonds from the poly(allylamine)phosphazene backbone via a thiol-ene photoreaction. Due to these two functionalities, a “vesicle”-like self-assembled structure was formed in the reaction medium, which could be then utilized for the microparticle preparation. The influence of NaOH during the synthesis was shown to affect the size and the wall thickness of the microparticles. This effect may enhance the possibilities to tailor such microparticles for drug delivery purposes or for future controlled release of other substances, such as drugs, fragrances, or anticorrosive pigments.

## 1. Introduction

Vesicles that form a hollow-like structure by a bilayer membrane are of major interest for their potential encapsulation and release of guest species in applications such as biomedical [1], catalysis [2], energy storage [3,4], and environmental remediation [5]. These micro/nanomaterials present a significant large surface area due to the existence of the hollow cavity, while the density is much lower than their solid counterparts due to the abundant inner void space with the same composition and size. 

Several studies have described the formation of this kind of hollow particles using amphiphilic copolymers that in selective solvents can generate a variety of morphologies, such as spherical particles, cylindrical rods, or vesicles [6,7]. However, in constructing amphiphilic polymers with different hydrophilic/hydrophobic ratios, the well-established polyphosphazenes (PPz) should be considered strong prospects [8]. These are tunable polymers the backbone of which contains alternating nitrogen and phosphorus atoms. Each phosphorus atom can be substituted with organic or inorganic groups according to their features and degradability [9]. A highly suitable strategy for end-group or side-group functionalization is the use of click chemistry, specifically, photo-initiated thiol-ene coupling reaction [10]. A previous study in our lab accomplished the silane functionalization of polyphosphazene using this approach [11]. 

Organic–inorganic hybrid vesicles have more stability compared to pure organic polymer vesicles [12]. In particular, silica-based organic–inorganic hybrid hollow micro- and nanoparticles are the most interesting class [13]. There are two other main methods for preparing hollow particles, namely, the hard- and soft-templating approaches [7]. However, for practical use, a third method—the self-templating strategy and subsequent stabilization by sol-gel chemistry—may be preferable due to the simplified synthesis, reduced production costs, and ease of scaling up [14,15]. With this method, the presence of reactive, repeating units within the polymeric structure, such as alkoxysilanes, may induce chemical crosslinking, and thus the fixation of the vesicle shape, yielding a silica inorganic structure [16].

Following this self-templating strategy, we report a novel route to prepare hollow microparticles using a novel reactive copolymer, “vesicle”-like silane-polyphosphazene, without the need to add further classical silanes to fix the vesicular morphology. Amphiphilic polyphosphazenes containing trimethoxysilanes and long alkyl groups with mutable ratios were synthesized by means of a two-step thiol-ene reaction. The influence of the preparation conditions and the amounts of gelation base catalysts on the formation of these novel hybrid vesicles was studied. One advantage of this method is that it allowed the formation of hollow microparticles while avoiding the need for additional templates, or a step to remove the surfactant.

## 2. Materials and Methods

*Chemicals:* Anhydrous diethylether (p.a.) was purchased from VWR; PCl3, allylamine, 2,2-dimethoxy-2-phenylacetophenone (DMPA) (99%), dodecanethiol, lithiumbis(trimethylsilyl)amide (LiN(SiMe3)2 (97%), dichlorodiphenylphosphorane (Ph3Cl2) (95%), hexachlorocyclotriphosphazene (99%), and (3-mercaptopropyl)trimethoxysilane (95%) were ordered from Sigma Aldrich; SOCl2 (99%) were ordered from Fluka; anhydrous DCM and THF were ordered from Alfa Aesar; triethylamine and ethanol (p.a) were ordered from Merck. Deuterated chloroform (CDCl3) from Eurisotop was used for NMR measurements. Triethylamine was distilled before usage; the other chemicals and solvents were purchased and used as received.

*Characterization methods:* For measuring ^1^H- and ^31^P-NMR spectra, a Bruker Avance III 300 was utilized (Bruker BioSpin GmbH, Rheinstetten, Germany). The ^1^H-NMR spectra were taken at 300 MHz using CDCl_3_ or MeOD as solvent. The ^31^P-NMR spectra were measured at 121 MHz. Dynamic light scattering (DLS) measurements were carried out using a Zetasizer Nano ZSP (Malvern Instruments, Worcestershire, UK) in ethanol (0.5 mg of sample in 1.5 mL of solvent) in glass cuvettes, previously sonicated for 30 min. The DLS measurements by intensity, volume, and number were performed six times at 20 °C, without filtration. For Fourier-transform infrared spectroscopy (FTIR) a Spectrum 100 FTIR spectrometer (PerkinElmer, Buckinghamshire, UK) was utilized with attenuated total reflection (ATR), and the measurements were performed in the range from 600 to 4000 cm^−1^. SEM images were obtained with a Jeol 6400 (Jeol, Peabody, MA, USA). Thermogravimetric analyses (TGA) were performed with a TGA/PerkinElmer Q5000 (TA instruments, New Castle, DE, USA), with a heating range from 70 to 900 °C using platinum pans and a heating rate of 10 °C min^−1^ under a flow rate of 25 mL min^−1^ of N_2_.

*Synthesis of Cl_3_PNTMS monomer:* The monomer was prepared according to literature procedures (as seen in Figure S1) [17] and the chemical structure was confirmed by ^1^H- and ^31^P-NMR spectroscopy. ^1^H-NMR (300 MHz, CDCl_3_, δ/ppm): 0.18 (s, 9H) (see Figure S2); ^31^P-NMR (300 MHz, CDCl_3_, δ/ppm): −54.5 (see Figure S3).

*Synthesis of the poly[bis(allylamino)phosphazene]:* The precursor was prepared as previously reported by our group [11] (Figure S4) and the chemical structure confirmed by ^1^H- and ^31^P-NMR spectroscopy. ^1^H-NMR (300 MHz, CDCl_3_, δ/ppm): 7.5 (m, 15H); 5.89 (m, 2H); 5.05 (m, 4H); 3.56 (m, 4H) (see Figure S5); ^31^P NMR (300 MHz, CDCl_3_, δ/ppm): 17.41; 10.02 (see Figure S6). 

**Figure 1:**
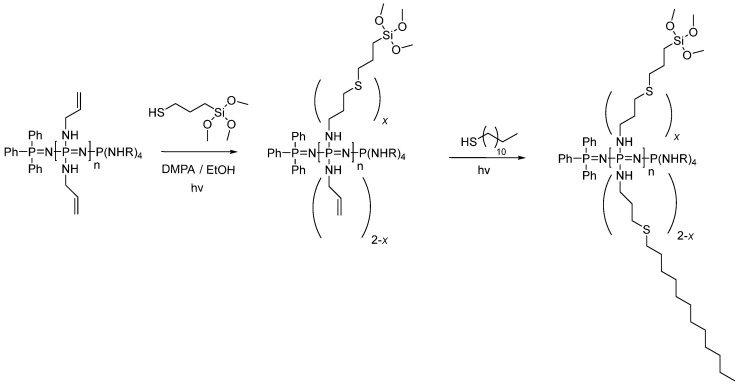
Reaction scheme of the silane-derived polyphosphazene (PPz).

*Synthesis of silane-derived polyphosphazene (UV reaction):* For the synthesis of the silane-derived polymer, two different thiols were used for the thiol-ene photoreaction (Figure 1). First, (3-mercaptopropyl)trimethoxysilane was added to the polymer backbone, as reported by our group [11], and in the second step, dodecanethiol was attached. For the synthesis of the linear silane-derived phosphazene, poly[bis(allylamino)phosphazene] (0.5 g, 3.18 mmol) was dissolved in 25 mL degassed THF. One equivalent of (3-mercaptopropyl)trimethoxysilane (0.59 mL, 3.18 mmol) was added, followed by DMPA (0.25 g, 0.98 mmol, 0.3 eq.). The solution was transferred to the UV reactor and the reactor was flushed with argon. Then, the mixture was irradiated with UV light for 15 min while stirring. Subsequently, the mixture was cooled with an ice bath for 15 min. These irradiation and cooling steps were repeated four times. Afterwards, the second thiol, a dodecanethiol, (0.79 mL, 3.32 mmol, 1 eq.) was added, and the mixture was again irradiated with UV light for 15 min and afterwards cooled using an ice bath. The same procedure as described before (irradiation and cooling) was repeated four times. To avoid crosslinking, the solvent was not evaporated, and the polymer was directly used for microparticle preparation. Quantitative yields were obtained, and the polymer was characterized by ^1^H- and ^31^P-NMR spectroscopy. ^1^H-NMR (300 MHz, CDCl_3_, δ/ppm): 0.76–0.71 (m, 2H); 0.88–0.84 (m, 3H); 1.24 (s, 20H); 1.83 (s, 4H); 2.54–2.47 (m, 10H); 2.70–2.53 (m, 4H); 3.55 (s, 9H) (see Figure S7); ^31^P-NMR (300 MHz, CDCl_3_, δ/ppm):13.50; 6.37 (see Figure S8). Mn (calculated by NMR, n~5) = ca. 40,000 g mol^−1^.

*Synthesis of silane-based microparticles withpolyphosphazene:* A mixture of 120 mL Milli-Q water and 30 mL EtOH (water:EtOH 80:20 *v*/*v*%) was stirred in a beaker and heated up to 80 °C. Then, the previously prepared silane-derived polyphosphazene (0.5 mL, 0.06 mmol) was added dropwise with different dropping rates (the first 0.2 mL was added at 50 μL/s, followed by the other 0.3 mL added at 100 μL/s), and the mixture immediately turned turbid. After dropwise addition of different amounts of 2 M NaOH solution (8, 6 and 4 mL), the formation of particles was visible to the naked eye (so called A1–C2, respectively). The mixture was stirred vigorously (1200 rpm) for one hour at 80 °C and was then cooled to room temperature while stirring. Two different methods were applied to obtain the microparticles, including centrifugation, on the one hand, and vacuum filtration followed by centrifugation, on the other hand, achieving 84 wt% and 16 wt% for small and big hollow microparticles, respectively. In both cases, the particles were washed with Milli-Q water until reaching pH 7. In Table 1, the reaction conditions (amounts of base, method of addition of phosphazene solution and the method to obtain the microparticles) are listed. The microparticles were dried overnight under air conditions (obtaining ca. 28 mg for A1 and C1 and ca. 23 mg for B1).

## 3. Results and Discussion

### 3.1. Synthesis of Silane-Derived Amphiphilic Polymer

For the synthesis of the polymer, a phosphine-mediated living cationic polymerization was utilized according to literature procedures [11,18] to give a poly(allylamine)phosphazene with five repeat units. A thiol-ene photoreaction was chosen to attach 1 eq. of (3-mercaptopropyl)trimethoxysilane to the double bonds [11]. Approximately half the double bonds reacted with the silane-containing molecule. The remaining allyl moieties were then substituted with an excess of dodecane-1-thiol to combine both functionalities in one polymer with an approximate ratio of 1:1. The side stemming from the (3-mercaptopropyl)trimethoxysilane were hydrophilic and those from the dodecanethiol were hydrophobic, so this functionalized phosphazene could act as a “vesicle” due to the combination of both hydrophilic and hydrophobic units in the same polymer [19,20]. This behavior is commonly observed in amphiphilic polyphosphazenes.

In the ^1^H-NMR spectrum after UV reaction, no peaks between 5 and 6 ppm, assigned to the protons from double bonds, were visible (see Figure S7). The absence of these protons was an indication of complete conversion via the UV photoreaction utilizing these two thiols. The complete assignment of all protons is difficult due to large EtOH peaks visible in the ^1^H-NMR spectrum, because of the EtOH peaks overlap with peaks from the molecule, which makes the integration of the peaks really complicated. Nevertheless, the EtOH could not be removed entirely after the UV reaction due to the tendency of the silane groups to crosslink. The silane-derived phosphazene solution was directly used for the microparticles’ preparation. The ^31^P-NMR spectrum further confirmed the structure of the product (Figure S8). 

### 3.2. Preparation of Silica Microparticles

**Figure 2:**
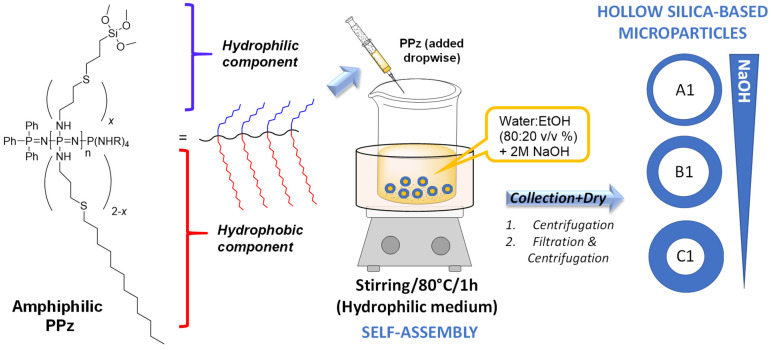
Scheme of preparation of hollow silica microcapsules (A1–C1) without any surfactant using the silane-derived polyphosphazene with different amounts of base. Microparticles are collected by either centrifugation (1) or filtration and centrifugation (2) followed by drying.

Organic–inorganic hybrid hollow microparticles with robust polysilsesquioxane walls can be produced by the formation of a vesicle after the copolymer is put in contact with water/methanol solution and the introduction of a small amount of catalyst [21]. The amphiphilic polyphosphazene was then directly used for the surfactant-free preparation of hollow microparticles (Figure 2). The silane groups were necessary to form the silica network during the sol-gel reaction. Then, the microparticles were collected (either by centrifugation or by vacuum filtration followed by centrifugation steps) and washed to a neutral pH with Milli-Q water. The morphology (sizes of particles and holes) of the microparticles was characterized by scanning and transmission electron microscopy (SEM and TEM) and their size distribution was determined by dynamic light scattering (DLS). 

During the preparation, the previously synthesized polyphosphazene precursor was added dropwise to an aqueous solution at 80 °C utilizing a syringe with a needle. Using this method of preparation, materials with non-uniformly distributed diameters may be obtained due to the possible inhomogeneous manual addition of the polymer solution, which may result in a large size distribution. In an attempt to improve that method, a syringe pump was utilized for a more accurate addition of the silane source. However, with this method no particles were obtained due to a possible crosslinking and unintended particle formation in the needle caused by the water vapor formed during the addition of the phosphazene solution at 80 °C. 

**Figure 3:**
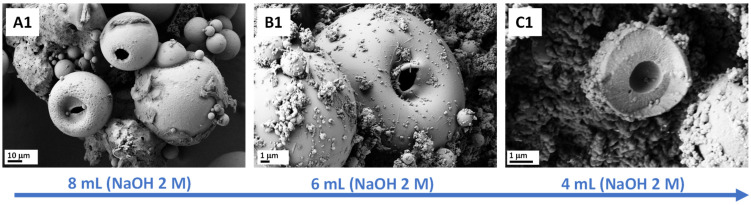
SEM images of hollow silica microparticles obtained using different amounts of base, with 8, 6 and 4 mL of NaOH (2M) yielding to microparticles (**A1**), (**B1**) and (**C1**) respectively, scale 10 µm (left (**A1**)) and 1 µm (center (**B1**) and right (**C1**)).

According to the SEM images (Figure 3), broad ranges of silica-based microparticle sizes (1–90 μm) with hollow morphology were successfully prepared using this sol-gel method, utilizing only a phosphazene containing two functionalities, without the addition of extra silica sources, e.g., tetraethyl orthosilicate (TEOS) or surfactants. Interestingly, broken particles were also visible during SEM measurements (see Figure 3(C1)), demonstrating their hollow features. A reason for this could be that their thin pore wall, compared to the size of the microparticle, did not survive the high vacuum conditions within the microscope. This breakage, however, facilitate the observation of hollow morphology of these microparticles. From the SEM images, various kinds of material were observed, such as small, spherical, compact silica particles and large microparticles. The sizes of the large microparticles were measured up to 90 μm for A1. According to the literature [22], the more base is added, the larger the particles become, as observed in the SEM images. 

This method was modified to aid microparticle formation by altering the reaction conditions. In the “traditional” sol-gel reaction, the silane source is added to a water/base mixture containing a surfactant and the directly formed particles can be collected by centrifugation after the reaction. In our case, the polyphosphazene solution (our silane source) was added dropwise to a water/ethanol mixture, before the addition of a base, leading to the direct reaction of the silane groups, which resulted in a turbid solution, observable by the naked eye. Here, the addition of a base as a gelation catalyst is necessary to catalyze the formation of particles/microparticles through a condensation reaction, fixing the vesicular morphology [23,24]. Without adding a base, the silane groups may hydrolyze, but no particles/microparticles form that can be collected from the solution, due to the missing condensation step. In some cases, the addition of base leads to agglomeration of the material, which could be correlated to the amount of base used. The more base that is utilized for microparticle preparation, the larger the formed aggregates become. Therefore, to identify the ideal concentrations of water, ethanol, base and silane source for successful microparticle formation, different amounts of base were selected (2 M NaOH solution, using 8, 6 and 4 mL) (see Table 1) in order to investigate the influence of the base on microparticle formation. The hydrodynamic diameters (D_h_) obtained from DLS measurements from A1–C1 by intensity are shown in Table 1 (see D_h_ by volume and number in the Supporting Information). It is worth noting that DLS gave the average particle size of all the suspended particles (aggregates and larger particles tend to precipitate so they were not measured), mainly measuring the smaller (800–160 nm) microparticles; this differs from the SEM method, which measures whole dry sample. DLS from C2 was not measured. 

**Figure 4:**
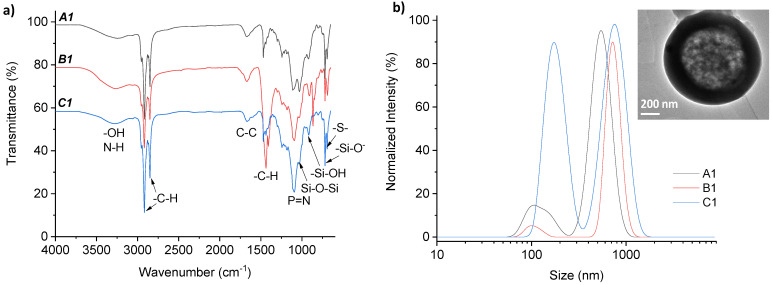
(**a**) FT-IR spectra and (**b**) DLS measurements (hydrodynamic diameter by normalized intensity) of the three hollow microparticles A1, B1, and C1; Inset (**b**) TEM image from B1 microparticles, scale 200 nm.

As already reported [22], the amount of base (2 M NaOH solution) used can influence the particle size such that the more base is added, the larger the formed particles are. Hence, different amounts of base were added during microparticle preparation in order to influence the size of the formed microparticles and obtain a more homogeneous particle size distribution. As seen from the data collected in Table 1, mainly microparticles with large (>800 nm) and small (<800 nm) diameters with hollow structures were found, with a range of inner hole sizes, demonstrating the influence of the amount of base on hollow-structure formation. However, the formation of nanoparticles (<160 nm) was not avoided. Neither the 8 mL of base for preparing hollow microparticles (A1) nor the 4 mL 2 M NaOH solution for preparing microparticles (C1) yielded homogeneous-sized particles. Interestingly, for the case of microparticles prepared using 4 mL 2 M NaOH solution (C1), smaller cavities and smaller microparticles were formed during the silanization process, as already observed with the formation of A1–B1, thus confirming a trend in formation of the hollow microparticles’ features according to the amount of base used during the synthesis. The C1 microparticles were between 1.1 to 3.8 μm in size, which is much smaller than the materials obtained in A1 (<90 μm) or B1 (<14.5 μm). From Figure 3, it can be seen that C1 particles had much thicker pore walls (~1.5–2.6 μm) and consequently smaller inner cavities (~1.75–4 μm) in comparison with A1 and B1, which gave a ratio of wall thickness to hole diameter of 1:1.1 for C1, 1:3 for B1, and 1:8 for A1 (determined from the SEM images of A1 and C1 in the Supporting Information, Figure S9, and from the TEM image for B1, Figure 4b). In general, it was observed that the pore wall thickness may be influenced by the amount of base used, leading to the conclusion that a lower amount of base results in thicker pore walls.

The chemical structure of these three hollow microparticles was analyzed by FTIR (see Figure 4a), where the spectra of all the samples were similar, as expected. The peaks at 1435 and 1096 cm^−1^ were assigned to the P=N of the polyphosphazene, which may further demonstrate the composition of the microparticles. The formation of the Si–O–Si network was detected by the characteristic peak overlapping with the broad peak at 1032 cm^−1^. The peaks at 915 and 720 cm^−1^ were assigned to –SiOH, along with the broad peak at 3280 cm^−1^ from the surface –OH groups. Additionally, in the three cases of A1, B1, and C1, no signal at 2560 cm^−1^ was observed, which indicated no presence of unreacted −SH groups, and thus the peak at 689 cm^−1^ was attributed to the C–S–C vibration from the full conversion reaction of the alkoxysilane source (3-mercaptopropyl)-trimethoxysilane to form thioether units during the thiol-ene click UV reaction. The two intense peaks between 2900 and 2800 cm^−1^ and the peak at 1435 cm^−1^ are highly characteristic of the symmetric and asymmetric stretching and scissoring vibrations of the methylene groups (–CH), together with C–C at 1670 cm^−1^, due to the presence of long alkyl chains from the polyphosphazene molecule.

The organic content was also determined by thermogravimetric analysis (see TGA spectra in Figure S10 in Supplementary Materials), where a similar tendency was observed for the three types of microparticles (a total weight loss of 66.9, 62.2, and 68.4% was determined for A1, B1 and C1, respectively). As seen in all three TGAs, a negligible first weight loss was detected below 120 °C (<1%) corresponding to the evaporation of residual small molecules, such as water and ethanol, possibly adsorbed onto the material. The materials started to lose weight around 150 °C, with the most pronounced weight loss beginning at around 200 °C, centered between 218 and 230 °C, where the samples lost almost half their weight by 250 °C. After this temperature range, another decomposition temperature was detected between 300 and 350 °C. From 600 °C to the end of the measurement, at 900 °C, a minor final weight loss was detected (2–5%) due to the possible whole decomposition of phosphazene moieties, leaving a residual weight of ca. 30% corresponding to the condensation of silanols from the silica network. 

The hydrodynamic diameters of the microparticles were measured by DLS (average by intensity of at least 4 independent measurements; see size distribution by intensity in Figure 4b, and volume and number in Figure S11, as well as average hydrodynamic diameters from two family sizes of particles collected in Table S1). The hollow silica-based particles showed similar bimodal distributions at this concentration, which clearly indicated that two types of particles were formed: micro- and nanoparticles (635 ± 139 nm and 152 ± 28 nm for A1, 653 ± 97 nm and 87 ± 8 nm for B1, and 734 ± 158 nm and 158 ± 27 nm for C1, respectively, see data in Table 1). Interestingly, slightly bigger particles (100 nm larger) were obtained for C1. From these DLS data on volume, the ratio of total mixture of particles between larger (>800 nm) and smaller (800–160 nm) hollow microparticles was determined to be 5–8% and 93–95% for A1-B1 and 28% and 72% for C1.

Transmission electron microscopy (TEM) was utilized to confirm the spherical, hollow morphology of the synthesized silica–phosphazene hybrid microparticles, and in particular, B1 was selected for capturing the size of particles and inner hollowed cavity, since it was the most homogeneously obtained material (according to the DLS results). TEM measurements showed similar formation of spherical particles with centered cavities (shown in Figure 4b, inset image) with an average diameter of 1000 nm, in agreement with hydrodynamic diameters obtained from DLS measurements (ca. 650 nm for B1, as seen Figure 4b). 

**Figure 5:**
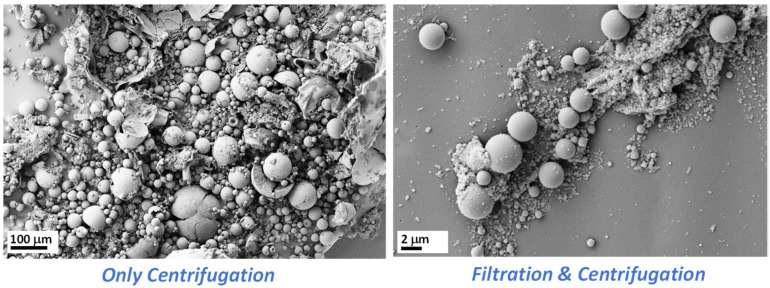
SEM images of hollow silica microparticles C1 (**left**) and C2 (**right**), collected by centrifugation alone or by initially applying vacuum filtration followed by centrifugation. Scale: 100 µm (left) and 2 µm (right).

In order to separate the large microparticles and the small microparticles, the large microparticles were collected by physical separation—in this case, by vacuum filtration—while the remaining suspended small microparticles were collected by centrifugation, yielding the particles referred to here as C2. The same type of particles (C1 and C2) were washed with Milli-Q water until they reached pH = 7 and were again characterized using SEM (see Figure 5). The only difference between these two materials (C1 and C2) was the method of collecting the particles (centrifugation of the whole mixture after synthesis in the case of C1 microparticles and vacuum filtration for the collection of the larger C2 hollow particles), which does not affect the particle’s morphology. In the SEM image from Figure 5, it can be clearly observed that many small spherical particles were collected (800–160 nm), although the presence of a few microparticles with diameters between 0.8–4 μm was still not avoided. The addition of vacuum filtration may be a promising, though not optimal, method to separate this type of particle by size. 

## 4. Conclusions

Silica hollow microparticles were prepared using only a silane-derived polyphosphazene as the precursor for their formation, without an additional silane source. Due to both polyphosphazene functionalities, a “vesicle”-like self-assembled structure was formed in the reaction medium which could be then utilized for microparticle preparation without the need for an extra surfactant. The success of this sol-gel process in preparing hollow silica microparticles based solely on the polyphosphazene precursor was analyzed by means of SEM and TEM, FT-IR, TGA and DLS. The SEM demonstrated that these novel microparticles could be prepared but that small nanoparticles were also formed. Two methods were utilized to solve this issue. In one case, a physical approach was used to separate the two materials by vacuum filtration to separate the large microparticles and obtain small particles by centrifugation. In the other, different reaction conditions were utilized to investigate the influence of various amounts of base on microparticle formation. From the SEM images and DLS measurements, it was noticeable that the microparticles formed after adding only 4 mL of 2 M NaOH solution were smaller compared to the microparticles formed when larger amounts (6 or 8 mL) of base were used. Furthermore, it could be observed that pore wall thickness could be correlated to the amount of base used, where less base used, the thicker the wall and the smaller the cavity obtained. This simple method of producing phosphazene-based silica hollow microparticles through a self-templating strategy may be of interest for different encapsulating purposes and release of different guest species in a variety of applications.

## Figures and Tables

**Table 1:** Method conditions and characterization of the silica-based particles.

Hollow Silica Microparticles	PPz Solution (mL)	2 M NaOH (mL)	PPz Addition Method	Particles Collection Method	D_h-DLS_ (nm)	D_SEM_ (μm)
A1	0.5	8	Dropwise	Centrifugation	635 ± 139; 152 ± 28	<90
B1	0.5	6	Dropwise	Centrifugation	653 ± 97; 87 ± 8	<14.5
C1	0.5	4	Dropwise	Centrifugation	734 ± 158; 158 ± 27	<4
C2	0.5	4	Dropwise	Filtration and centrifugation	-	<4

## Supplementary Materials

The following supporting information can be downloaded at: https://www.mdpi.com/article/10.3390/ma15144763/s1, All the NMR spectra are shown in the ESI, available online, Figure S1. Synthesis of the Cl_3_PNSiMe_3_ monomer. Figures S2 and S3: ^1^H-NMR and ^31^P-NMR spectrum of the monomer Cl_3_PNTMS in CDCl_3._ Figure S4: Synthesis of linear poly[bis(allylamino)phosphazene] 6 (n~5). Figures S5 and S6: ^1^H-NMR and ^31^P-NMR spectrum of the poly[bis(allylamino)phosphazene] (n~5) in CDCl_3_; Figures S7 and S8: ^1^H-NMR and ^31^P-NMR spectrum of the silane-derived polyphosphazene in CDCl_3_. Figure S9: SEM images of hollow silica microparticles obtained using different amounts of base, with 8 and 4 mL of NaOH (2M) yielding microparticles A1 and C1 respectively, scale 10 µm (left (A1)) and 1 µm (right (C1)). Figure S10: TGA of the microparticles. Figure S11: DLS of silica-based particles A1–C1 in normalized intensity, volume, and number (%). Table S1: DLS size distribution (hydrodynamic diameter average) of the silica-based microparticles A1–C1 collected after centrifugation.
