# Supplementary material for: Hollow Silica Microparticles Based on Amphiphilic Polyphosphazenes
Source: Materials (Basel). 2022 Jul 7;15(14):4763. doi: 10.3390/ma15144763 (PMC9318875; doi:10.3390/ma15144763)
Supplement: Supplementary file 1 [file materials-15-04763-s001.zip › materials-1753896-supplementary_changes.pdf]

Supporting Information

Hollow silica microparticles based on amphiphilc polyphosphazenes

Yolanda Salinas*, Vanessa Poscher, Oliver Brüggemann, and Ian Teasdale

Institute of Polymer Chemistry and Linz Institute of Technology, Johannes Kepler University Linz, Altenberger Straße 69, 4040 Linz, Austria.

*Correspondence E-mail: yolanda.salinas@jku.at

Synthesis and NMR characterization of Cl_3PNTMS

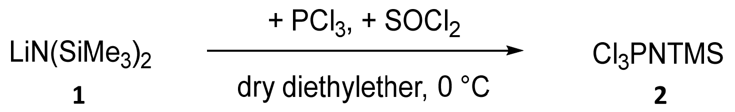

Figure S1. Synthesis of the $Cl_3PNSiMe_3$ monomer.

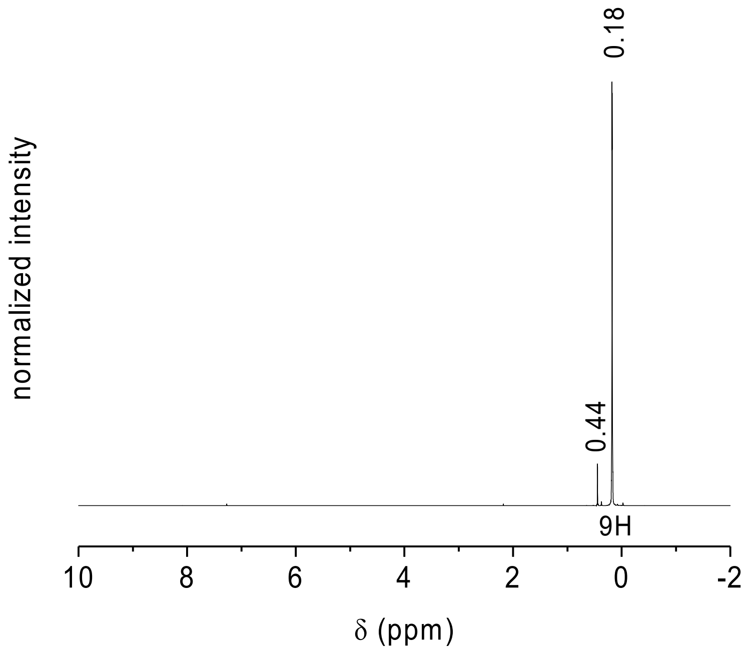

Figure S2. ^{1}H-NMR spectrum of **2** (monomer Cl_3PNTMS) in $CDCl_3$.

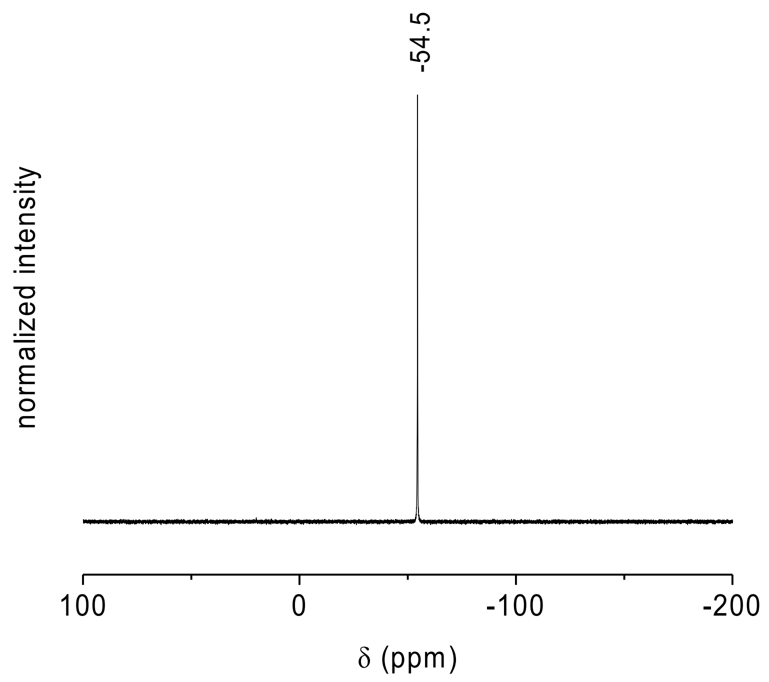

Figure S3. ^{31}P-NMR spectrum of **2** (monomer Cl_3PNTMS) in $CDCl_3$.

Synthesis and NMR characterization of poly[bis(allylamino)phosphazene]

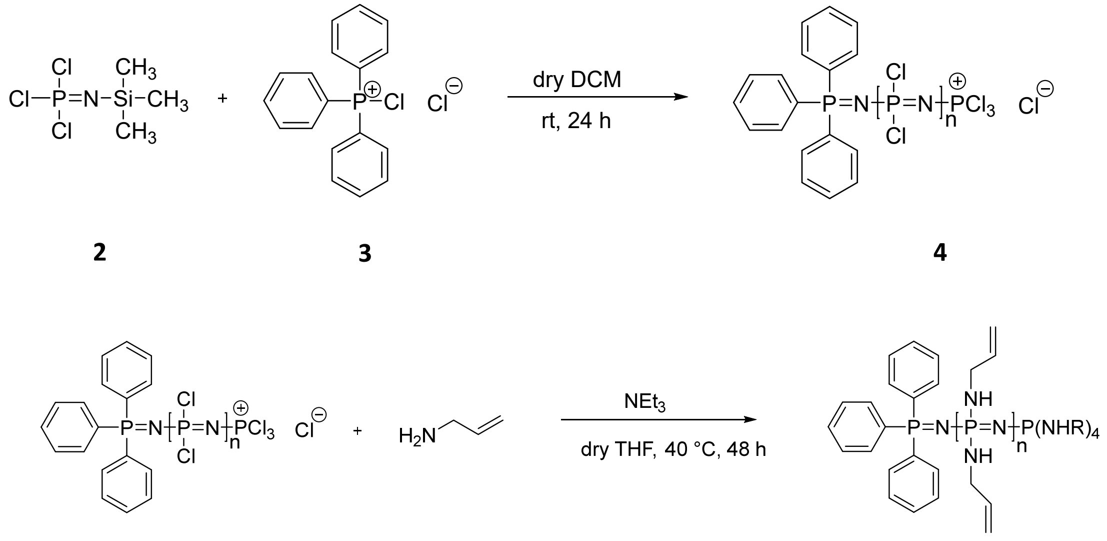

Figure S4. Synthesis of linear poly[bis(allylamino)phosphazene] **6** (n ~ 5).

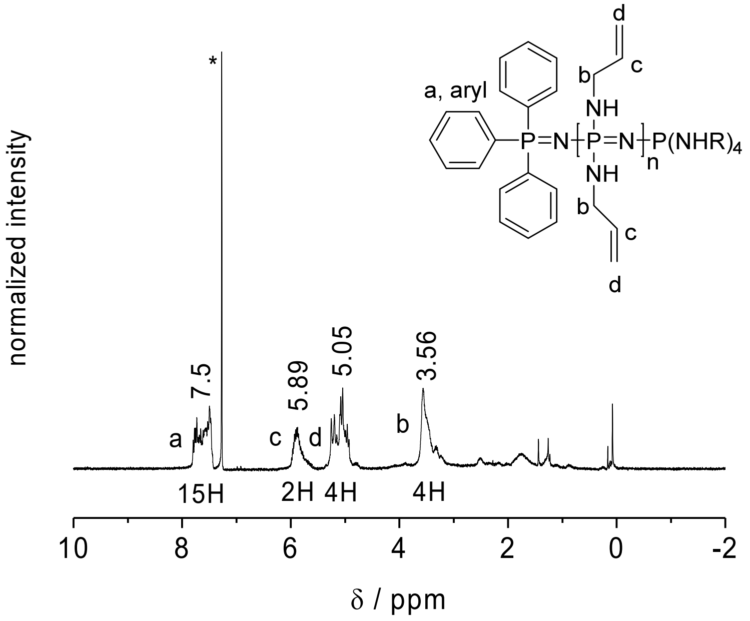

Figure S5. ^{1}H-NMR spectrum of **6** (n ~ 5) in $CDCl_3$ (*).

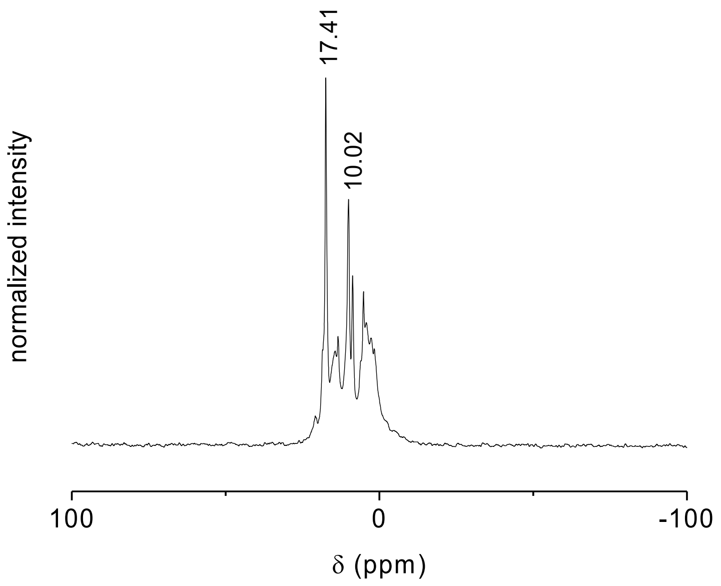

Figure S6. ^{31}P-NMR spectrum of **6** (n ~ 5) in $CDCl_3$.

Synthesis and NMR characterization of the silane derived linear polyphosphazene

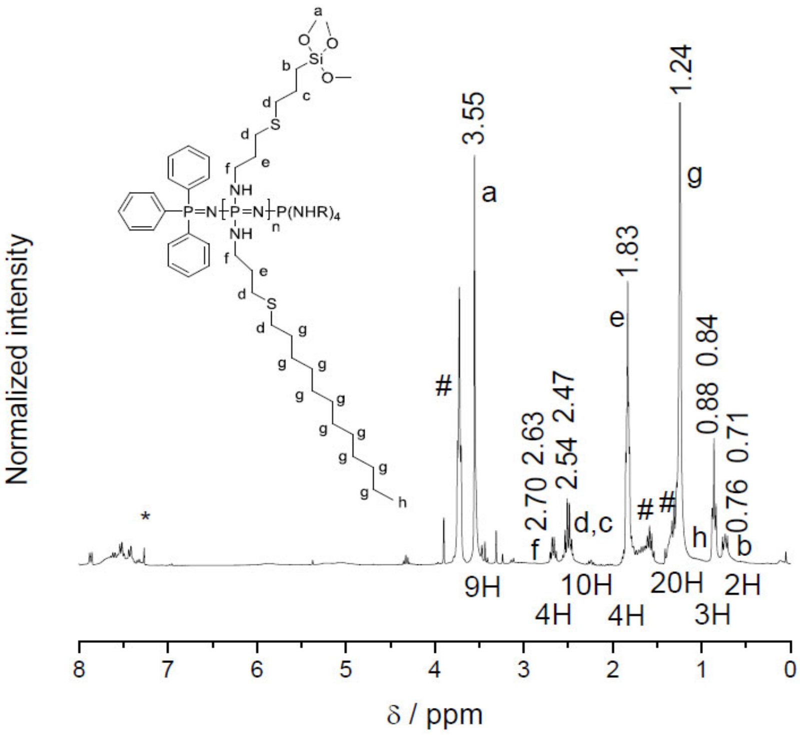

Figure S7. ^{1}H-NMR spectrum of the silane derived polyphosphazene precursor after UV reaction using two different thiols in $CDCl_3$ (*). EtOH peaks (#) present and overlapping with peaks from the silane precursor.

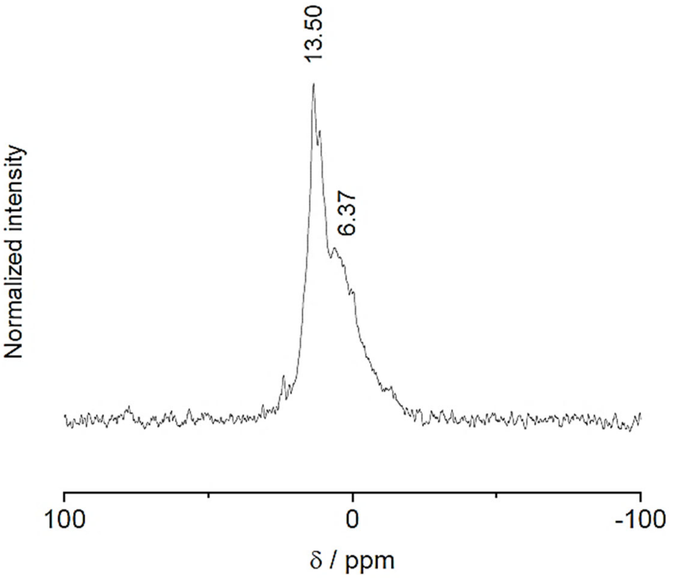

Figure S8. ^{31}P-NMR spectrum of the silane derived phosphazene precursor after UV reaction using two different thiols.

Microparticles characterization

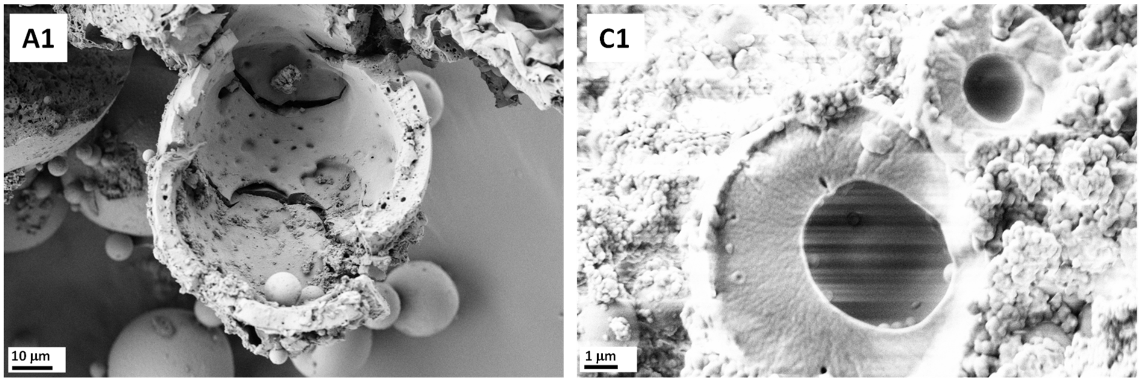

Figure S9. SEM images of hollow silica microparticles obtained using different amounts of base, with 8 and 4 mL of NaOH (2M) yielding to microparticles A1 and C1, respectively, scale 10 µm (left (A1)) and 1 µm (and right (C1)).

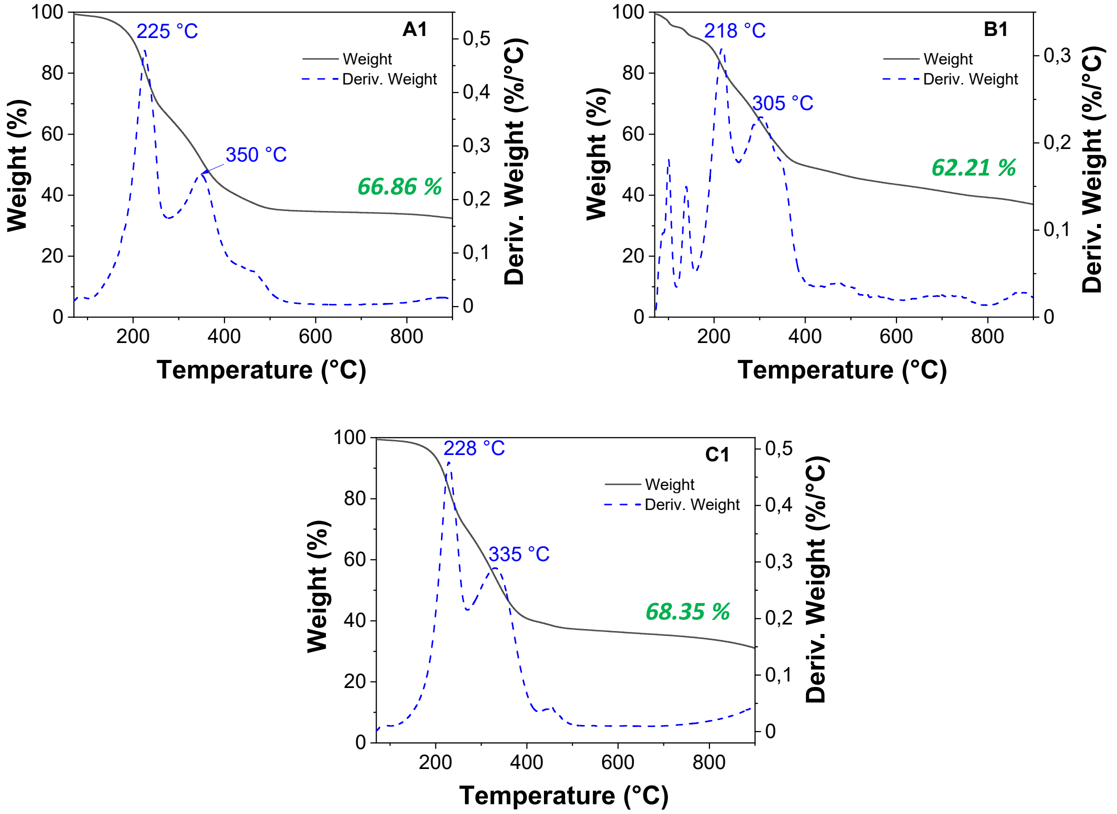

Figure S10. TGA of the silica-based particles

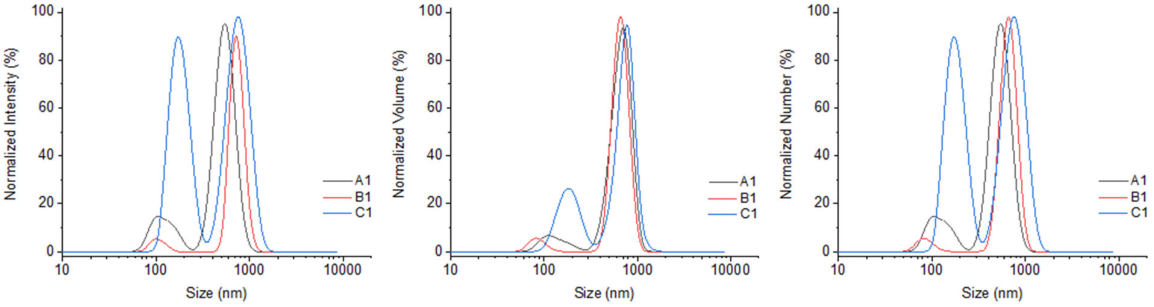

Figure S11. DLS of silica-based particles A1-C1 in intensity, volume and number (%)

Table S1. DLS size distribution (hydrodynamic diameter average) of the silica-based microparticles A1-C1 collected after centrifugation

	**Intensity**		**Volume**		**Number**	
**Particles**	D_{h1} (nm)	D_{h2} (nm)	D_{h1} (nm)	D_{h2} (nm)	D_{h1} (nm)	D_{h2} (nm)
**A1**	635±139	152±28	680±147	150±33	598±144	134±28
**B1**	653±97	87±8	691±123	86±12	646±120	84±11
**C1**	734±158	158±27	740±142	157±33	676±142	144±29
